# Stability of Metronidazole and Its Complexes with Silver(I) Salts under Various Stress Conditions

**DOI:** 10.3390/molecules26123582

**Published:** 2021-06-11

**Authors:** Małgorzata Starek, Monika Dąbrowska, Joanna Chebda, Dominik Żyro, Justyn Ochocki

**Affiliations:** 1 Department of Inorganic and Analytical Chemistry, Faculty of Pharmacy, Jagiellonian University Medical College, Medyczna 9, 30-688 Kraków, Poland; m.starek@uj.edu.pl (M.S.); monika.1.dabrowska@uj.edu.pl (M.D.); jmalina328@gmail.com (J.C.); 2 Department of Bioinorganic Chemistry, Chair of Medicinal Chemistry, Faculty of Pharmacy, Medical University of Lodz, Muszyńskiego 1, 90-151 Łódź, Poland; dominik.zyro@umed.lodz.pl

**Keywords:** metronidazole, TLC-densitometry, validation of the method, stability testing, silver(I) complex

## Abstract

Metronidazole is a drug widely used in the prevention and treatment of bacterial infections. Due to its possibility of the formation of stable metal complexes, it was decided to broaden its activity spectrum by introducing the silver(I) coordination compounds i.e., [Ag(MTZ)_2_NO_3_] and [(Ag(MTZ)_2_)_2_]SO_4_, which have significant antibacterial properties. The paper presents a description of a new qualitative and quantitative analysis of metronidazole in bulk and possible pharmaceutical preparations by thin-layer chromatography with densitometric detection. Optimal separation conditions were selected, and the analytical procedure was validated according to the ICH guidelines. The obtained data indicate that the method is sufficiently sensitive, precise, and accurate. The stability of the metronidazole solutions obtained from tablets, pure metronidazole, and its silver(I) complexes was tested. The research was carried out in various environments, at different temperatures, in H_2_O_2_ solution, and during exposure to radiation (UV, sunlight). The greatest degradation was found in the alkaline environment and at higher temperatures. The silver(I) complexes exhibited relatively high stability under analyzed conditions that are higher than standard metronidazole solutions and tablets. The observations were confirmed by the kinetic and thermodynamic analysis. The described studies of new metronidazole silver(I) complexes increase the potential for their application in infections both in humans and animals.

## 1. Introduction

**Figure 1:**
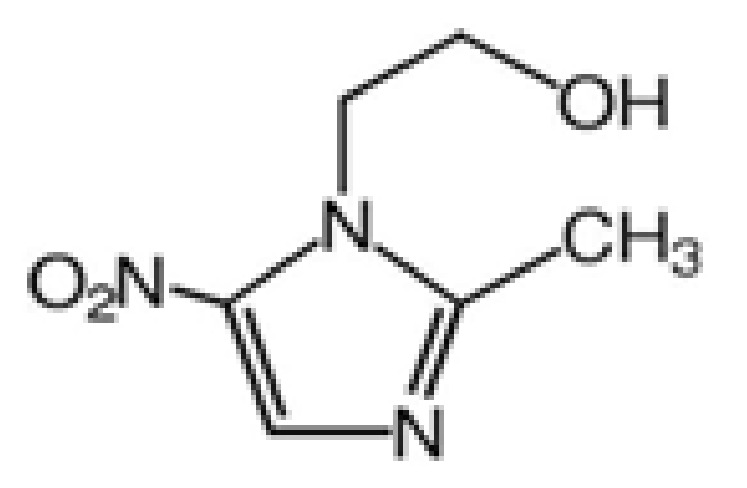
Chemical structure of metronidazole (MTZ) [3].

Metronidazole (MTZ, 2-metyl-5-nitro-1H-imidazole-1-ethanol; Figure 1) is a derivative of 5-nitroimidazole. It exhibits the greatest activity against anaerobic Gram-negative bacteria but has no activity against aerobic bacteria [1]. As a chemotherapeutic agent, it is used to treat infections caused by most anaerobic bacteria and protozoa, such as *Trichomonas vaginalis*, *Entamoeba histolytica*, or *Giardia lamblia*. It is used in the therapy of trichomoniasis of the genitourinary system, amoebiasis, giardiasis, treatment of infections such as *Clostridium, Eubacterium, Bacteroides, Fusobacterium,* and *Gardnerella vaginalis*. It is widely used also in the prevention of post-operative infections and therapeutically in conditions caused by anaerobic bacteria, such as sepsis, pneumonia, brain abscess, peritonitis, osteomyelitis, puerperal fever, or bedsores and ulcers of the lower limbs [2]. In addition, in combination therapy with other drugs, it is used in the eradication of *Helicobacter pylori* [3]. The mechanism of action of metronidazole is to reduce the nitro group in an environment with low oxygen content. Free nitro radicals are formed, which damage the DNA chain and lead to the destruction of the microbial cell [4].

The formulations of metronidazole may have general action achieved by oral or parenteral (intravenous in the form of injection solutions and infusion) or topical application to the skin, vaginally, rectally in suitable forms such as vaginal tablets, suppositories, cream, gel, or ointment [5]. In anaerobic bacterial infection, the daily doses usually used in adults and children over 12 years are from 750 to 1500 mg, whereas for children from 8 weeks to 12 years, the dose is usually 20–30 mg/kg. The drug should not be used for more than 7 days. After oral administration in the fed state of 250 mg, 500 mg, or 2 g of healthy volunteers, the maximum plasma concentration is achieved between 1 and 3 h and is 4.6–6.5 g/mL, 11.5–13 µg/mL, and 30–45 µg/mL. The rate of absorption and the maximum serum concentration of MTZ are dependent on the meal. About 20% of metronidazole is bound to proteins. MTZ is extensively absorbed from the gastrointestinal tract. It is easily distributed to all tissues, including the central nervous system, bones, breast milk, and sperm. It also crosses the placental barrier. In people with normal liver and kidney function, the biological half-life is between 6 and 8 h [3].

The primary site of metronidazole metabolism is the liver, where it undergoes glucuronic acid conjugation, oxidation, and hydroxylation. As a result, 2-hydroxymetronidazole is formed as the main metabolite, which also has antimicrobial activity. MTZ is eliminated by the kidneys (60–80%) and feces (6–15%). Its metabolites are dyes that are easily soluble in water, which is why the therapy may change urine to red-brown [3]. The drug often causes stomach and intestinal disorders in the form of nausea, vomiting, diarrhea, metallic taste in the mouth, or abdominal pain. Rare side effects include nervous system disorders (convulsions, encephalopathy, ataxia, headaches) and hematological disorders (leukopenia, thrombocytopenia, neutropenia, agranulocytosis, and pancytopenia). There have also been rare cases of fungal infections of the reproductive system, muscle pain, increased liver enzymes, rash, itching, redness, and blurred vision. After consuming alcohol, metronidazole may cause a disulfiram reaction, which is characterized by nausea, vomiting, heat bursts, and increased heart rate. It may also increase the effect of anticoagulants, e.g., warfarin. Drugs that affect the microsomal enzymes of the liver can accelerate the excretion of metronidazole, reducing its concentration in the blood (phenytoin, phenobarbital) or increase its half-life, e.g., cimetidine, which consequently increases the risk of side effects from the nervous system. MTZ belongs to class B according to the drug classification developed by the FDA in terms of safety in pregnancy, which means that animal studies have not shown harmful effects on the fetus, but the effects on human pregnancy have not been confirmed. Due to the penetration of the placental barrier and potentially mutagenic effects in animal studies, it is a drug prohibited in the first trimester of pregnancy, while in subsequent trimesters, it can be used only and exclusively when the possible benefits of taking outweigh the risk of adverse effects on the child [2,3].

MTZ is one of the chemical compounds that has the ability to form stable complexes with metals. The literature describes the synthesis and characteristics of stable complexes of MTZ with iron (in a molar ratio of 1:3), manganese, copper, cobalt, and nickel (1:2) [6]. In addition, the greater photostability of the drug in combination with sodium urate was demonstrated compared to free MTZ [7]. The use of the MTZ ester inclusion complex, as benzoate, together with β-cyclodextrin (in a 1:1.5 molar ratio) resulted in greater solubility, improved physical stability of the drug, and inhibition of the transformation of its anhydrous form into monohydrate, occurring in aqueous suspensions [8]. The MTZ complex with copper, ruthenium, and gold have also been studied. The complex with the copper showed the greatest activity during in vitro study against *Entamoeba histolytica* [9]. The combinations of MTZ with nucleic acids have been studied in mammals, bacteria, and phages under various conditions. Previous reduction of the drug with sodium dithionite was considered to be the most beneficial. It has been shown that there is then the formation of a covalent bond of the studied drug with cytosine and guanine. The resulting combination has an effect on both the antimicrobial and mutagenic activity of MTZ [10].

Since MTZ acts on anaerobic bacteria, research has been undertaken to combine it with silver to obtain a broader spectrum of activity. The idea of creating complex connections is associated with the antimicrobial effect of silver(I) compounds. Silver(I) nitrate is used in medicine, in the form of drops, which reduce the incidence of eye infections in newborns. Colloidal silver solutions are also used which, bound to proteins, show less irritant effects. Silver(I) combinations with drugs such as sulfadiazine are used to obtain a broader spectrum of activity in the treatment of burns, ulcers, or various types of wounds [11,12]. Two types of silver-containing dressings were also developed. The first was a combination of dressings containing metallic silver with a layer of activated carbon, which is used in the treatment of wounds, both oozing and dry, while the second was a dressing made of non-woven fabric coated with a substance, such as silver(I) sulfate, which allows the release of silver ions into the wound environment [13]. The method of obtaining complexes built of silver oxidation state +1, coordinated by metronidazole with appropriate anions of acids from the group of oxygen inorganic acids that are difficult to oxidize, e.g., HClO_3_, HClO_5_, H_2_SO_4_, and HNO_3_, were also described. Anions of tetrafluoroboric acid and halogenated organic sulfonic acids or halogenated carboxylic acid derivatives, e.g., trifluoroacetic acid, can also be used for the complex synthesis. It is important that the silver(I) salt used for the synthesis is at least partially soluble in the reaction medium. Therefore, silver(I) nitrate, chlorate, trifluoracetate, tetrafluoroborate, and methanesulfonate were used in the previous work. The synthesis was carried out in an aqueous ethanol solvent by mixing metronidazole with the corresponding salts in a 2:1 molar ratio at 60 °C in the absence of light [14]. The synthesis of the complex compound of metronidazole with silver(I) nitrate was modified for a one-step process in an aqueous environment [15]. Then, the resulting mixture was subjected to evaporation in vacuo and slow crystallization at room temperature [14]. The resulting complexes were characterized by ESI-MS, IR, NMR spectroscopy, and elemental analysis. The structure of each complex was also determined using X-rays.

In the next stage, the authors conducted studies on the stability of the complexes obtained under the influence of light. Cotton flakes were soaked with aqueous solutions of complexes and, for comparison, solutions of appropriate silver(I) salts. Test samples were left at room temperature with access of light and air. Their darkness was visually checked during 5 days. As a result of the study, much better stability of the complexes was obtained than the corresponding silver(I) salts, which began to darken after 4 h, and after 24 h, they were completely dark. Flakes soaked in solutions of complexes began to change color only after 48 h (beige color) [14]. In further studies, the light stability of the silver(I) complex of metronidazole was as assessed [16].

The antifungal and antibacterial activity of the complexes of the silver(I) with sulfadiazine, corresponding silver(I) salts, and with MTZ against microorganisms, such as *Staphylococcus epidermidis, Staphylococcus aureus, Escherichia coli, Pseudomonas aeruginosa, Proteus hauseri,* and *Candida albicans*, were compared. Parameters characterizing the antimicrobial activity of the tested compounds were described, i.e., MIC (minimal inhibitory concentration) and MBC or MFC (minimal bactericidal or fungicidal concentration). Free MTZ showed no activity against tested microorganisms. However, all the obtained complexes showed greater antibacterial activity against *Staphylococcus epidermidis* than individual silver(I) salts and silver(I) complex with sulfadiazine. The complex containing methanesulfonic anion showed the best antibacterial activity. Almost all tested complex combinations (except for the NO_3_^−^ containing counter-ion) also showed greater activity against *Pseudomonas aeruginosa* than reference compounds. Complexes with BF_4_^−^ and ClO_4_^−^ counter-ions revealed the greatest activity against *Escherichia coli* and *Proteus hauseri*. However, better antifungal activity against *Candida albicans*, compared to reference compounds, was demonstrated by complexes containing BF_4_^−^ and SO_3_CH_3_^−^ counter-ions [14]. The described studies of new silver(I) complexes with metronidazole may provide treatment infections both in humans and animals in the future. In addition to their beneficial antimicrobial properties, silver(I) in these complexes also improves the solubility of the entire complex in water and thus in body fluids, and therefore, they can be added to various types of medical materials. In addition, these complexes can be used externally in dressing materials, gels, or ointments, without causing the undesirable effect of discoloration.

In the available literature, a reverse phase system with gradient elution was used to determine MTZ by high performance liquid chromatography (HPLC) and spectrophotometric detection [17,18,19]. The HPLC method with a gradient elution system and detection at 242 nm was also used to determine MTZ in the presence of other drugs (diloxanide, spiramycin, clioquinol) in tablets [20]. Thin layer chromatography (TLC) was used in the study of MTZ in mixtures with chemotherapeutic drugs and antibiotics, using TLC 60F_254_ silica gel plates and various mobile phases [4,21,22,23]. The assay of MTZ beside clotrimazole, mebeverine hydrochloride, and diloxanide furoate with densitometric detection has also been described [24,25]. The HPTLC method has also been used to simultaneously measure MTZ in the presence of spiramycin or miconazole nitrate with silica gel 60F_254_ plates and densitometric detection at 240 nm [26]. The literature describes also the assay of MTZ and miconazole nitrate by the GC method with nitrogen as the inert gas and flame ionization detector (FID) [27].

Stability testing of substances consists of exposing samples to numerous factors, which theoretically may cause its degradation. The test compound can be subjected to acid and base hydrolysis, oxidation, and photochemical or thermal degradation. The information on MTZ stability studies using various analytical methods [4,28,29,30,31,32,33,34] is presented in Table S1 in the Supplementary Materials.

An assessment of the MTZ stability in the form of a concentrated suspension (50 mg/mL), which masks the bitter taste of the drug, was also carried out HPLC. The study showed the high stability of obtained mixtures in acidic solutions (after 314 h at 50 °C, 83% MTZ remained), while in an alkaline environment, after 24 h, only 9.2% remained. [35]. The MTC content was analyzed by HPLC after a short-term stability test to evaluate the behavior of the formulation during transport and by a long-term stability study to analyze the storage conditions. The studies showed the high stability of MTZ during both short and long-term tests of the analyzed preparation [36]. Wu et al. analyzed the level of MTZ degradation under the influence of various factors, both in solid and liquid form. The study lasted 8 weeks, and the samples were analyzed using the HPLC method. The obtained results confirmed the high stability of MTZ in the analyzed preparation. The authors also carried out MTZ stability studies in solutions with other drugs such as tetracycline hydrochloride, famotidine, and CBS (colloidal bismuth citrate) [37].

Bacterial strains are constantly developing resistance to medications used in medicine. New drugs are very important in saving human health. The most obvious solution seems to be the discovery of new drugs, but the path to developing a new drug requires a huge investment. By giving new life to old drugs, the modulation role of existing chemotherapeutic agents can be increased. In our earlier work, we described the synthesis of the silver(I) complex with MTZ and the study of its cytotoxic activity against Balb/c3T3, HepG2, PANC-1, and 1.2B4 cells [15,16]. The paper presents clinical trials confirming the effectiveness of ocular rosacea treatment with new formulations of dosage forms, which are drops and ointment with the silver(I) metronidazole complex in prescription drug [38]. The formula for eye drops is Rp. silver nitrate 0.1 g, metronidazole 0.2 g, boric acid 0.2 g, water 9.5 g. For ointment, the formula is is Rp. silver nitrate 0.1 g, metronidazole 0.2 g, water q.s. (*quantum satis*), lanolin 2.0 g, petrolatum white to 10.0 g. The presented results suggest that the new complex may be an alternative treatment method in cases of ophthalmic complications of rosacea, including inflammation of the cornea, which is resistant to standard pharmacological treatment.

**Figure 2:**
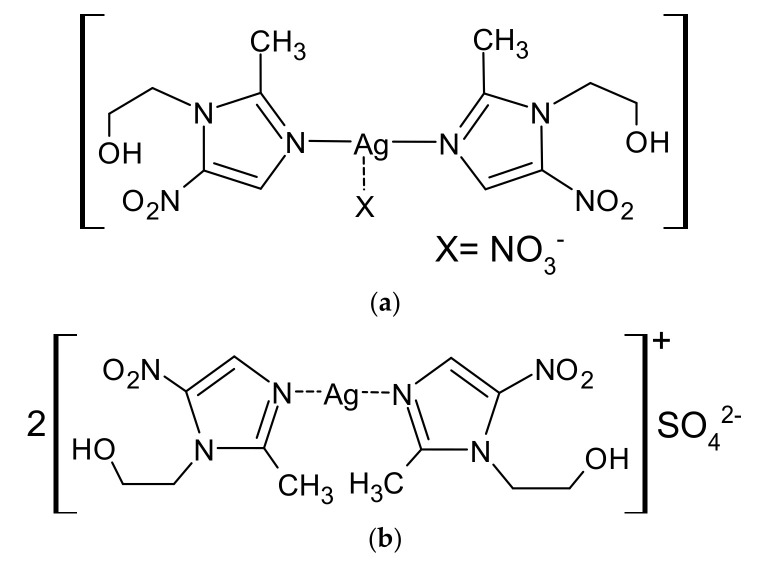
Complexes: I: metronidazole with silver(I) nitrate (**a**) and II: metronidazole with silver(I) sulfate (**b**).

The stability of drug substances and finished drug products is a key aspect of ensuring the quality of a drug and the safety of the therapy. Any analytical approach taking into account the minimization of the risk of degradation of the drug substance and the elimination of products with a potential toxic hazard is a priority in the registration and implementation proceedings. Thanks to technological progress, it is possible to constantly increase the quality control of medicinal products. Due to the wide use of MTZ and silver(I) in medicine and the development of a new formulation of dosage forms, the metronidazole complex with silver(I) nitrate, it was decided to analyze its stability under various environmental conditions. Complexes I and II used in our study are presented in Figure 2a,b.

## 2. Results

At the beginning of the present study, a method for the qualitative and quantitative determination of metronidazole by TLC with densitometric detection was developed. Based on the available literature, the conditions for MTZ analysis have been optimized alongside the components present in the solution. Finally, Silicagel 60F_254_ TLC chromatography plates and the mobile phase that consisted of ethyl acetate + methanol + ammonia 25% (15:5:0.5, *v/v/v*) were chosen. Densitometric detection was performed at the analytical wavelength λ = 297 nm, and the obtained retardation factor R_f_ for MTZ is 0.77. The developed analytical method was also validated, according to ICH guidelines, in terms of linearity, sensitivity, precision, and accuracy. Obtained values of individual parameters were collected in Table 1.

## 3. Discussion

Analyzing the above results, it can be concluded that the developed method shows the rectilinear relationship of the measured value on the concentration of the analyzed substance in the range of 2.8 to 4.6 µg of MTZ per spot. The calculated LOD and LOQ values indicate high sensitivity, and the RSD value of 0.71% indicates the high precision of the developed method. Based on the obtained peak area values, the percentage of MTZ recovery was calculated and is close to 100%, which also indicates the high accuracy of the used method.

Next, the developed analytical procedure was used to determine changes in MTZ content during stability tests in acid and alkaline environment, at elevated temperature (30, 60, 90 °C), 3% solution of hydrogen peroxide, and under the UV and solar radiation. During the analysis, peak area values for MTZ were recorded and converted into percentages of active substance in relation to respective peak areas obtained at t = 0. Tables S2–S5 (see Supplementary Materials) contain the values of substance content changes, which were obtained for the standard solution of MTZ, the metronidazole complexes with silver(I) ions, and the pharmaceutical preparation, respectively.

Based on the results obtained, it first can be stated that the rate of MTZ degradation is proportional to the increase of the temperature. In addition, the substance shows less stability in an alkaline environment than in an acidic one. The complete decomposition of MTZ in the presence of 3M NaOH occurs after 23 h at 30 °C, while at 60 °C and 90 °C, it occurs after only 1 h. The decreasing NaOH concentration increases stability, e.g., complete degradation in the presence of 0.5M NaOH occurs only at 60 °C after 23 h, while at 90 °C, it occurs after only 3 h of incubation. A decrease in solution stability is associated with an increase in acid concentration. During the examined period, the MTZ substance in acidic solutions did not degrade completely. In the 3M HCl environment after 23 h at 90 °C, 14.88% of the initial content remained. At 30 °C after 23 h in the presence of 0.1M HCl, 69.41% remained, while in the same conditions for the methanolic solution, this value was 70.81%.

Analyzing the stability of the complexes of MTZ with silver(I) ions, it can also be seen that it decreases with increasing temperature and under the influence of increasing pH. In solutions prepared in 3M NaOH at 60 and 90 °C, complete degradation takes place within an hour. Under the influence of lower base concentrations, e.g., 0.1M NaOH at 90 °C, complete decomposition occurred after 2 h. The complex also shows greater stability in an acidic environment. For complex I, after 23 h in the presence of 0.1M HCl, nearly 80% (60°) and 67% (90°) of metronidazole remained, while in methanolic solution, it was about 65 and 45%, respectively. On the other hand, complex II showed a slightly lower stability under the same conditions, i.e., 64 and 55%, respectively (62 and 43% for methanol solutions). The initial concentration of metronidazole did not change after treating the complex compound with acid or base, but reactions took place, resulting in the disintegration of the complex compound itself. However, this does not affect the determination of the metronidazole content in the sample. The action of HCl or NaOH on the complex compound gives the products of the molecule decomposition. It is silver(I) chloride in the case of HCl, and silver(I) oxide in the case of NaOH [39].

**Figure 3:**
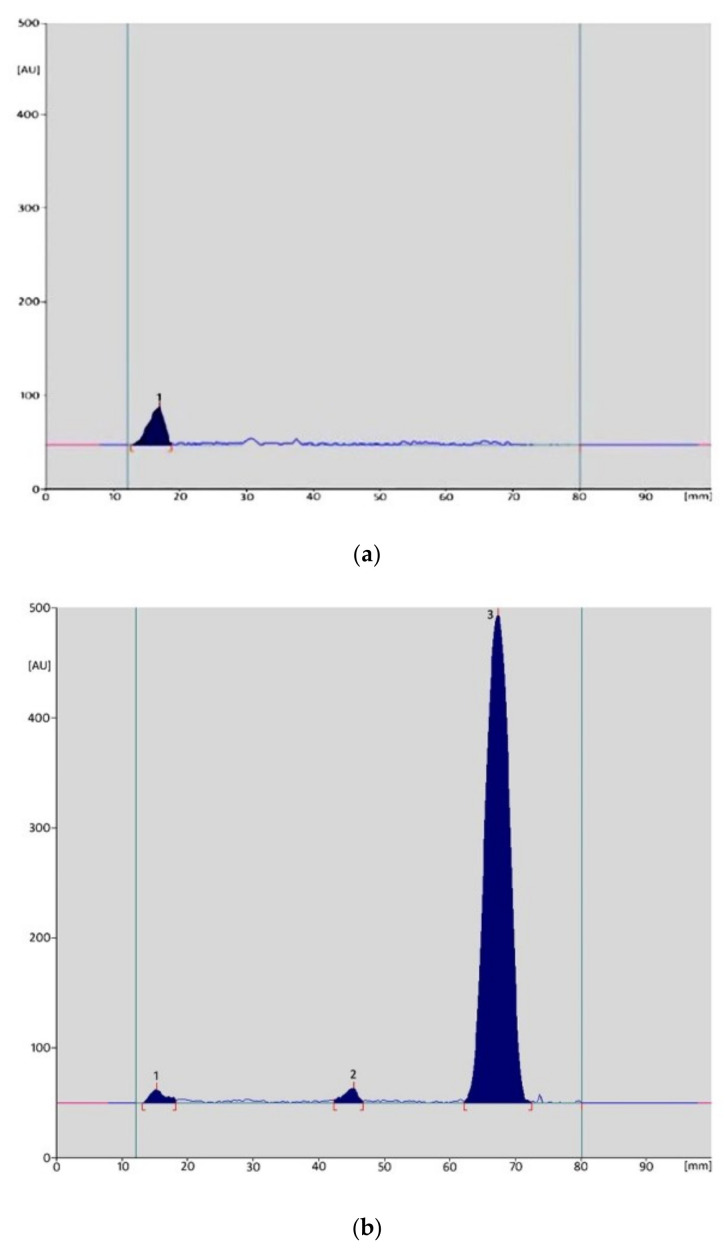
An example of densitograms obtained for MTZ standard substance solution in 1M HCl (**a**) and 1M NaOH (**b**) solutions after incubation at 30 °C for 23 h (peak 3—MTZ, peaks 1 and 2—degradation products).

Similar observations concern the effect of temperature on the stability of MTZ in the form of a finished product. The highest decomposition took place in a strongly alkaline environment, while MTZ showed the highest stability in the presence of 0.1M HCl. At 30, 60, and 90 °C, 88.33, 66.69, and 63.42% of substance remained, respectively. These values are higher than the analogous values obtained for the methanolic solution (respectively: 67.36, 59.74, and 46.06%). Figure 3 shows examples of densitograms obtained for solutions of the metronidazole standard substance, which were recorded during the test of its stability under various environmental conditions.

In the next stage, all forms containing MTZ (standard substance, complexes with silver(I) salts, tablets) were exposed to 3% H_2_O_2_ solution and elevated temperature. The obtained results (Table S6, see Supplementary Materials) indicate the relatively high stability of the substance under these conditions. The greatest decomposition took place at 90 °C, where 80.65% remained in the solution containing the reference substance, and 85.92, 40.26, and 90.56% remained in the complex I, II and drug solutions, respectively.

Stability tests of MTZ exposed to radiation simulating the solar and UV radiation of a specific wavelength (254 nm) were also carried out. The resulting percentages for substances under these conditions are summarized in Tables S7–S9 (see Supplementary Materials).

**Figure 4:**
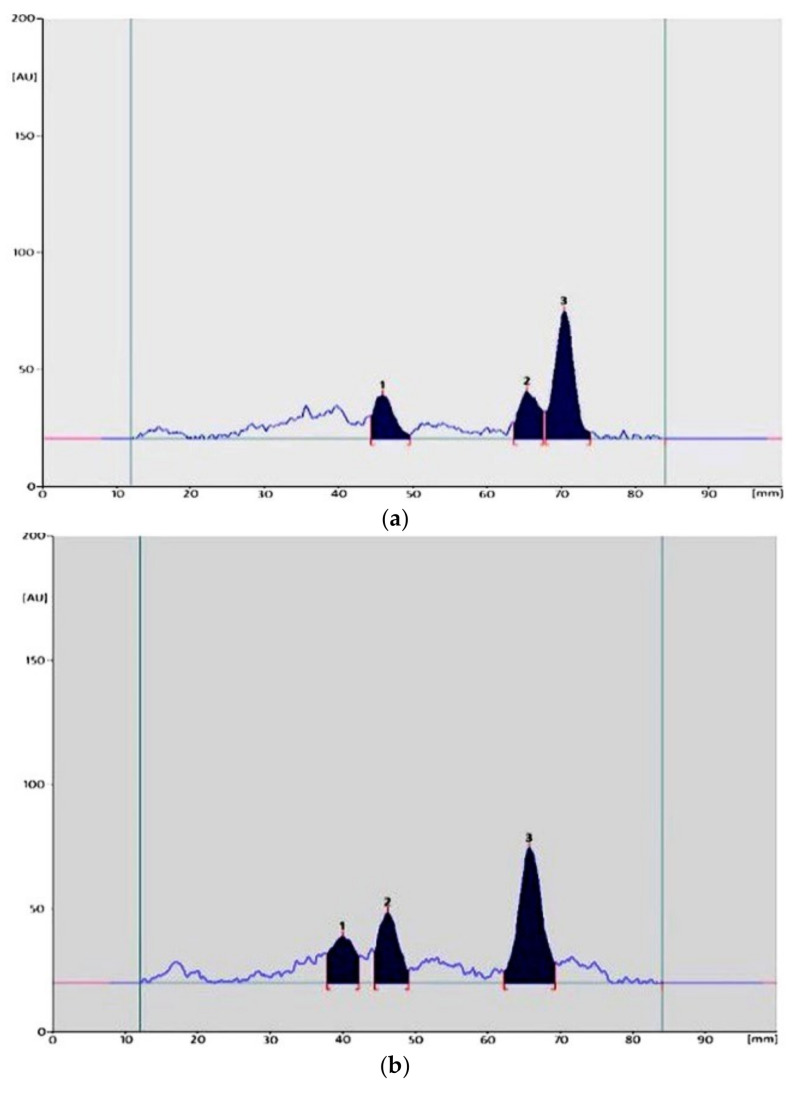

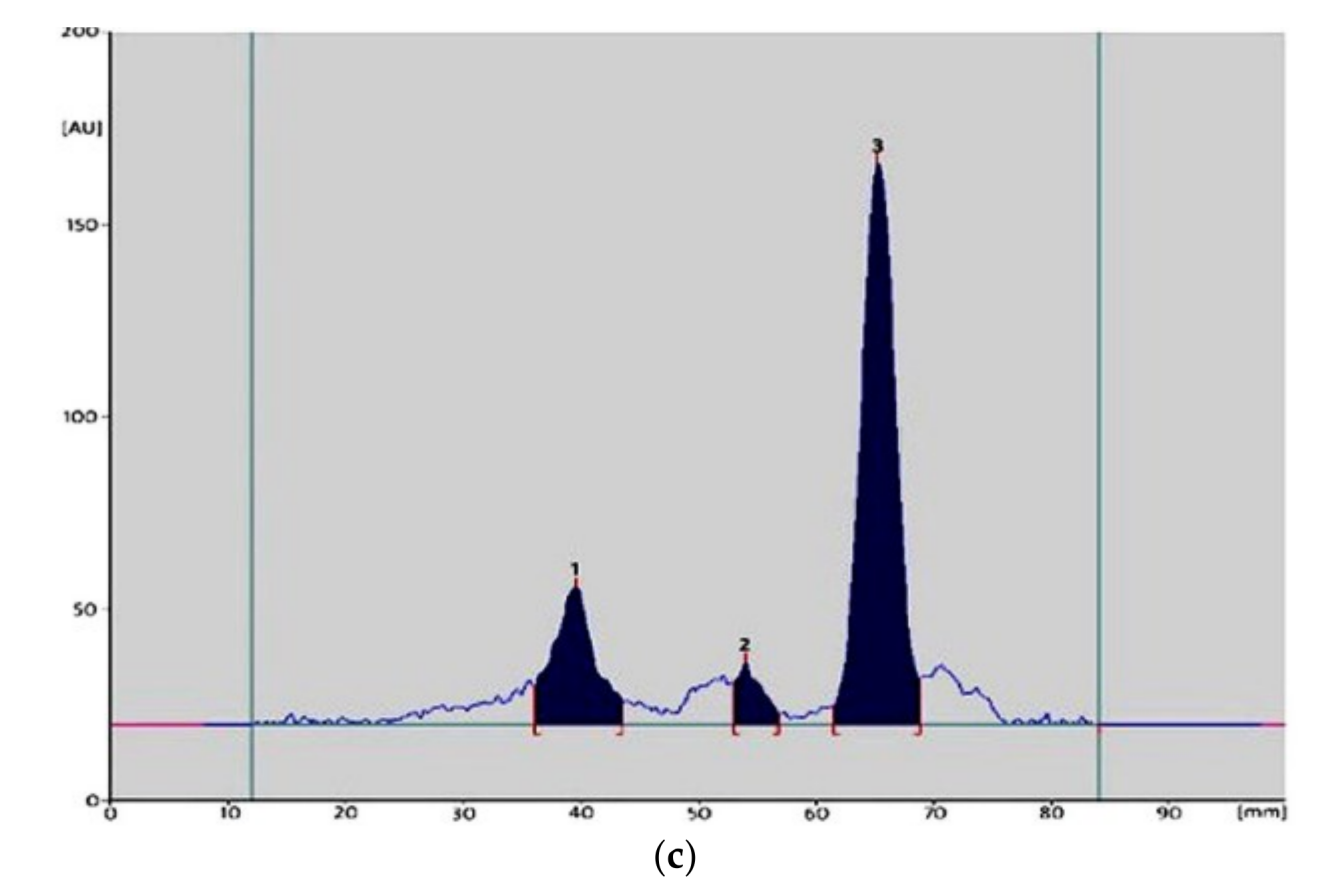
An example of densitograms obtained for MTZ standard substance (**a**), tablets (**b**), and complex I (**c**) solutions after 6 h of UV irradiation (peak 3—MTZ, peaks 1 and 2—degradation products).

Compared to the control, a large degradation of MTZ can be observed in the analyzed solutions (Figure 4). It can also be stated that the metronidazole complexes with silver(I) salts shows apparently greater stability than the standard and tablets solution. Its largest degradation can be seen only after 4 h of exposure, when a significant decrease in content was obtained. After this time, faster degradation of the substance took place in the solution of the reference substance, tablets, and complex II (11.23, 24.01, and 12.04%, respectively). While, after the last hour of exposure, they were almost completely decomposed (3.54, 9.76, and 0%). Then, a value of 21.38% was obtained for the solution of the complex I. When all forms of MTZ (substance, complexes, tablets) were exposed to sunlight irradiation, the obtained results show similar stability of the substance and the complex I, both in solutions and in the solid phase (above 76 and 88%, respectively). The lowest stability was observed for complex II, where 16 remained after 6 h of irradiation.

Next, natural logarithms from the percent concentration loss over time were calculated to determine the order of the MTZ degradation reaction under changing environmental conditions. Then, the curves of the dependence lnc on the duration (t) of the degradation factor were drawn. Due to the fact that the natural logarithm of the drug concentration is directly proportional to time, and the graph lnc = f(t) is characterized by a straight line relationship (Tables S10–S14, see Supplementary Materials), it was found that the degradation of MTZ under the influence of the analyzed factors occurs according to the kinetics for first-order reactions. Therefore, the values of the reaction rate constant (k) and times t_0.5_ and t_0.1_ were calculated [40], using the following formulae: k = 2.303(logc_1_−logc_2_)/(t_2_−t_1_), t_0.5_ = 0.635/k, t_0.1_ = 0.1053/k. The obtained results are collected in Table 2 and Table 3.

The calculated values of kinetic parameters confirm the relationship that the reaction rate constant increases with increasing pH and temperature, reaching extreme values for the solution of tablets containing MTZ: the lowest in 0.1M HCl (0.0057 h^−1^ at 30 °C) and the highest in 0.5M NaOH at 90 °C (3.5300 h^−1^). The rate constants for solutions of the MTZ standard substance are within the limits from 0.0152 (30 °C, methanolic solution) to 1.8500 h^−1^ (60 °C, 1M NaOH), while for complex I, the rate constants are from 0.0096 h^−1^ (30 °C at 0.5M HCl) to 2.7100 h^−1^ (60 °C, 1M NaOH). At the same time, the highest and lowest t_0.5_ values were found for the tablets’ solution, respectively 121.58 h (30 °C in 0.1M HCl) and 0.20 h (90 °C in 0.5M NaOH).

The highest k value for the MTZ degradation reaction in 3% hydrogen peroxide solutions was recorded for the standard substance at 90 °C (12.60 h). In the case of solutions treated with UV radiation, the calculated values far exceed the appropriate constants for the control samples, which confirms the ongoing process of MTZ degradation. A similar relationship can be found for solutions and solid phase substances exposed to radiation simulating sunlight.

To determine the dependence of the reaction rate on temperature (logk = f(1/T)) in the next step, the values of logk were calculated. Based on the obtained data (Arrhenius graphs), activation energy values (Ea) were calculated (E_a_ = −2.303 R(logk_1_−logk_2_)/(1/T_1_−1/T_2_), where R = 8.315 J/molK, k_2_ > k_1_, T_2_ > T_1_), and the obtained parameters are collected in Table 4.

As we know, the higher the activation energy, the more strongly the rate constant of a given reaction depends on the temperature. Based on the obtained results, it can be stated that the activation energy generally decreases with decreasing HCl and increasing NaOH concentration. However, in the presence of 3% hydrogen peroxide, it maintains relatively low values, the highest for the solution of the complex solutions, while the lowest are for the tablets solutions.

## 4. Materials and Methods

### 4.1. General Chemicals and Reagents

Metronidazole standard substance (MTZ; s.171852, exp. 06.2020) was purchased from Galfarm (Kraków, Poland). The silver(I) coordination compounds of metronidazole, in the form of [Ag(MTZ)_2_NO_3_] monomer (Complex I) [14] and [(Ag(MTZ)_2_)_2_SO_4_]·5H_2_O (Complex II) [41] were received from Department of Bioinorganic Chemistry, Chair of Medicinal Chemistry, Medical University of Lodz (Poland). Metronidazol Polpharma tablets containing of 500 mg of metronidazole (s.20918, exp. 09.2021) were purchased from the local pharmacy.

Methanol, ammonia 25%, acetone, glacial acetic acid, and hydrogen peroxide 30% were purchased from Chempur (Piekary Śląskie, Poland). Ethyl acetate, n-hexane, diethylamine, toluene, and chloroform were purchased from POCH (Gliwice, Poland). Butylamine was purchased from Fluka. All solvents were analytical grade.

### 4.2. Optimization of Analysis Conditions

**Figure 5:**
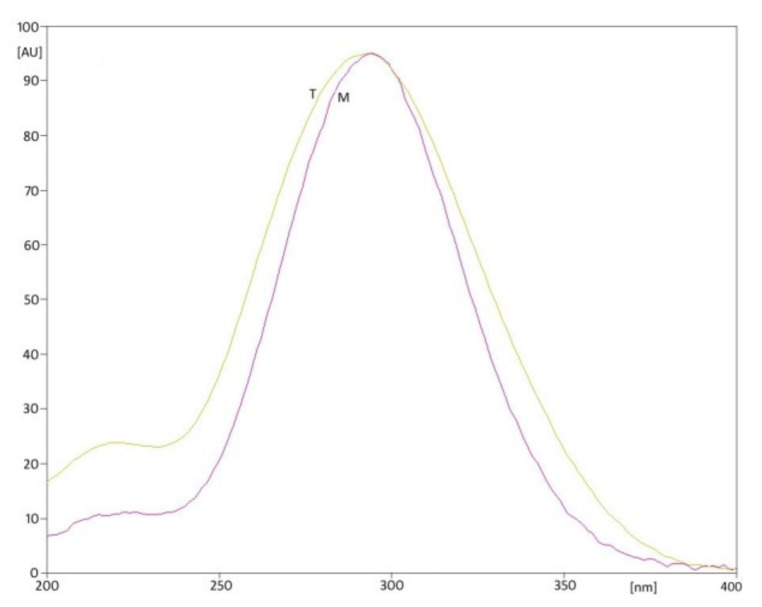
Absorption spectra for methanolic solutions of standard substance (M) and tablets containing metronidazole (T).

The selection of optimal analysis conditions was based on literature data on MTZ analysis using thin-layer chromatography. It was decided to conduct the analysis in a normal phase system, using chromatographic plates TLC Silicagel 60F_254_ (art. 1.05554, Merck, Germany) as the stationary phase. About 0.02% (*m/v*) solution of MTZ in methanol was applied to the plates in the form of bands (Linomat V; Camag, Muttenz, Switzerland) containing increasing amounts of the determined component. Then, the plates were developed in various mobile phases using solvent mixtures in various proportions. The obtained chromatograms were dried in the air and observed under a UV lamp (Cabinet 3; Camag, Muttenz, Switzerland) at wavelength λ = 254 nm. The best results were obtained using mobile phases: chloroform + acetone + glacial acetic acid (7.5:2.5:0.1, *v/v/v*), n-hexane + acetone + butylamine (7:3:0.6, *v/v/v*), and ethyl acetate + methanol + ammonia 25% (15:5:0.5, *v/v/v*). Chromatograms obtained under these conditions were scanned densitometrically (Scanner 3; Camag Muttenz, Switzerland). Absorption spectra for MTZ in the range 200–400 nm were also recorded directly from the chromatograms (Figure 5). Based on the obtained spectra, the analytical wavelength λ = 297 nm was selected, which was used for further analysis.

On the preliminary tests, the MTZ standard solution was exposed to an environment of various pH, using a hydrochloric acid and sodium hydroxide solutions (1M concentration). The samples were also subjected to an elevated temperature (80 °C) for 3 h. The obtained samples were applied to TLC Silicagel 60F_254_ chromatography plates and developed using 3 selected mobile phases.

Observations of MTZ stability under changing environmental conditions made during these studies indicated a decreasing content of active substance and the appearance of its degradation products (additional peaks registered on the densitograms). The best separation of the active substance from the degradation products was obtained using a mobile phase: ethyl acetate + methanol + ammonia 25% (15:5:0.5, *v/v/v*).

### 4.3. Validation of the Analytical Method

The validation of the MTZ determination conditions was carried out in accordance to the ICH guidelines [42]. This process involved determining the range of linearity, limit of detection and quantification, and the precision and accuracy of the developed method.

The range of linearity was determined using methanolic solutions of the MTZ standard substance in the concentration range from 1.89 to 5.12 µg/spot. A calibration curve was drawn between the amount of MTZ per spot and the respective peak areas obtained on densitograms, and then, regression parameters were determined.

Based on the determined linear regression parameters, the limits of detection (LOD) and quantification (LOQ) values were calculated. For this purpose, the obtained values of standard deviation (S) and slope of the straight line (a) were used, in accordance with the formulas: LOD = 3S/a and LOQ = 10S/a.

The precision of the method was tested using a 0.02% (*w/v*) MTZ standard solution. For this purpose, nine times by 10 μL of standard solution were applied to the chromatographic plates. Then, they were developed under the conditions described earlier; the peak areas on the obtained densitograms were recorded and subjected to statistical analysis (Statistica v.12, StatSoft, Tulsa, OK, USA).

The accuracy of the analytical method was expressed as a percentage of analyte recovery. For this purpose, the MTZ solution from tablets, the 0.02% (*w/v*) MTZ standard solution, and mixtures containing the preparation solution with the addition of the standard substance in the amount of 80, 100, and 120% were applied to the chromatographic plates. Plates were developed using the methodology described earlier and then scanned.

### 4.4. Stability Study

In the next stage, a 0.02% (*w/v*) methanolic solution of the MTZ standard substance was prepared, and solutions of the tested complexes (taking into account the molar mass of complex I, complex II, and metronidazole, i.e., 512.24, 1080.00, and 171.15 g/mol, respectively) and tablets (by weighing the appropriate amount of tablet weight) were calculated on the concentration of determined substance. The solutions prepared in this way were treated with HCl and NaOH solutions: 3M, 1M, 0.5M, and 0.1M. It was observed that a solution of the complex compound in a 1M, 0.5M, and 0.1M acidic medium shows opalescence, while that in an alkaline medium forms a black precipitate. Three sets of solutions as described above were prepared and stored at three temperatures: 30 °C, 60 °C, and 90 °C. During the time specified in the research plan (immediately after preparation, after 1, 2, 3, 4, and 23 h of incubation), the solutions were applied to chromatographic plates and analyzed in the conditions of the method described above.

Similarly, solutions containing MTZ in the form of a reference substance, complexes, and tablets were prepared, which were treated with a 3% solution of hydrogen peroxide. As before, prepared samples were stored at 30, 60, and 90 °C. Then, the solutions were applied to the plates at t = 0, 1, 2, 3, and 4 h, developed using the conditions (described above), and analyzed densitometrically.

In the next stage, the photostability of MTZ was checked by exposure (in closed quartz crucibles) to UV radiation (TUV lamp, 30 W, max. λ = 253.7 nm, amount of radiation: 1.244 mW/cm^2^ (energy 4.5870 J·s^−1^·(cm^2^)^−1^ after 1 h); Philips, Germany), and radiation simulating sunlight (300–800 nm; Suntest CPNplus Atlas, Wrocław, Poland) for 6 h (solutions) and 96 h (solid state). The obtained samples were analyzed according to the methodology described above. At the same time, a control test was performed in which the tested solutions were prepared in an analogous way, shielded from light, and stored under appropriate radiation sources.

## 5. Conclusions

In the presented study, the conditions for the determination of metronidazole by the TLC method with densitometric detection were optimized and the developed procedure was validated, confirming its usefulness in drug analysis. Then, the developed method was used to test the stability of metronidazole in solutions under changing environmental conditions. The research was carried out simultaneously for the solution of the reference substance MTZ and pharmaceutical preparations, such as tablets and for metronidazole silver(I) complexes. The solutions were subjected to increased temperature in acidic and alkaline solutions, in a 3% hydrogen peroxide solution and exposure to UV and sunlight. Based on the obtained results, kinetic parameters and activation energy values were calculated. All analyzed solutions showed the lowest stability at 90 °C, and a higher degree of degradation was observed in alkaline environment. MTZ showed relatively high stability in the presence of H_2_O_2_ and under the influence of sunlight irradiation (both in solution and in the solid state). On the other hand, during UV exposure, MTZ in the form of a complex I showed the highest stability. Control tests of raw materials and medicinal products are closely related to the need to monitor and assess the degree of degradation of the molecule. They identify two elements that form the basis of quality testing: durability and stability. The developed procedure leads to obtaining reliable data enabling the use of the acquired knowledge in the technology of drug production so that they are stable and safe. The demonstrated high stability of the new substances based on the complex of MTZ and silver(I) salt may indicate its effectiveness in therapy. The described studies of new combinations of MTZ confirm the increased possibilities of effective treatment of many diseases and ailments. TLC with densitometric detection is a fast, simple, and inexpensive analytical technique for the separation, identification, and quantification of various substances. It can be used in various fields of science, inter alia in laboratories for drug monitoring, quality control, and kinetic studies. Even though HPLC is the most commonly used method in stability studies, TLC has some advantages, such as the ability to perform analyses of several samples simultaneously, the subsequent storage of the plates, small sample volume, and short chromatography time per analysis, without the need for special sample preparation and costly solvents.

## Figures and Tables

**Table 1:** Parameters of the method validation.

Parameter		Values
Linearity		1.05—4.62 µg/spot P = 6854.9 + 3460.4·c r = 0.9830 S_a_ = 322.7 S_b_ = 999.2 S_e_ = 1044.1
LOQ		2.89 µg/spot
LOD		0.87 µg/spot
Range of the method		2.89—4.62 µg/spot
Precision		SD = 134.85 RSD% = 0.71
Accuracy	80%	$\bar{x}$ = 99.07 SD = 0.26 RSD% = 0.27
	100%	$\bar{x}$ = 99.89 SD = 0.86 RSD% = 0.86
	120%	$\bar{x}$ = 100.45 SD = 0.61 RSD% = 0.61

P—peak area; c—concentration; r—correlation coefficient; S_a_—standard deviation of the slope; S_b_—standard deviation of the intercept; S_e_—standard deviation of the estimation; $\bar{x}$—arithmetic mean; SD—standard deviation; RSD—relative standard deviation [%].

**Table 2:** Values of kinetic parameters for the MTZ solutions.

Solution	Parameter	MeOH	3M HCl	1M HCl	0.5M HCl	0.1M HCl	0.1M NaOH	0.5M NaOH	1M NaOH	3M NaOH	H_2_O_2_
30 °C											
Standard substance	k	0.0152	0.0226	0.0243	0.0187	0.0161	0.0239	0.0500	0.1900	0.3100	0.0200
	t_0.5_	45.59	30.66	28.52	37.06	43.04	29.00	13.86	3.65	2.24	34.65
	t_0.1_	6.93	4.66	4.34	5.64	6.55	4.41	2.12	0.55	0.34	5.27
Complex I	k	0.0135	0.0204	0.0335	0.0096	0.0100	0.0152	0.0491	0.1300	0.3750	0.0250
	t_0.5_	51.33	33.97	20.69	72.19	69.30	45.59	14.11	5.33	1.85	27.72
	t_0.1_	7.81	5.17	3.15	10.98	10.54	6.93	2.15	0.81	0.28	4.22
Complex II	k	0.0099	0.0217	0.0396	0.0096	0.0118	0.0224	0.1084	0.3894	0.6108	0.0259
	t_0.5_	69.93	31.98	17.52	72.26	58.68	30.98	6.39	1.78	1.13	26.79
	t_0.1_	10.63	4.86	2.66	10.98	8.92	4.71	0.97	0.27	0.17	4.07
Tablets	k	0.0174	0.0383	0.0435	0.0513	0.0057	0.0213	0.0574	0.3725	0.3800	0.0225
	t_0.5_	39.83	18.09	15.93	13.51	121.58	32.54	12.07	1.86	1.82	30.80
	t_0.1_	6.06	2.75	2.42	2.05	18.49	4.95	1.84	0.28	0.28	4.68
60 °C											
Standard substance	k	0.0261	0.0674	0.0352	0.0230	0.0213	0.1361	0.3100	1.8500	-	0.0375
	t_0.5_	26.55	10.28	19.69	30.13	32.54	5.09	2.24	0.37	-	18.48
	t_0.1_	4.04	1.56	2.99	4.58	4.95	0.77	0.34	0.06	-	2.81
Complex I	k	0.0191	0.0657	0.0439	0.0204	0.0109	0.0813	0.3350	2.7100	-	0.0275
	t_0.5_	36.28	10.55	15.79	33.97	63.58	8.52	2.07	0.26	-	25.20
	t_0.1_	5.52	1.60	2.40	5.17	9.67	1.30	0.31	0.04	-	3.83
Complex II	k	0.0205	0.0698	0.0457	0.0448	0.0191	0.1306	0.8442	2.1347	-	0.1127
	t_0.5_	33.82	9.94	15.17	15.49	36.29	5.31	0.82	0.32	-	6.15
	t_0.1_	5.14	1.51	2.30	2.35	5.51	0.81	0.12	0.05	-	0.93
Tablets	k	0.0226	0.0691	0.0522	0.0657	0.0178	0.2000	1.1200	2.53	-	0.0250
	t_0.5_	30.66	10.03	13.28	10.55	38.93	3.47	0.62	0.27	-	27.72
	t_0.1_	4.66	1.53	2.02	1.60	5.92	0.53	0.09	0.04	-	4.22
90 °C											
Standard substance	k	0.0439	0.0830	0.0652	0.0287	0.0261	0.9900	1.7500	-	-	0.0550
	t_0.5_	15.79	8.35	10.63	24.15	26.55	0.70	0.40	-	-	12.60
	t_0.1_	2.40	1.27	1.62	3.67	4.04	0.11	0.06	-	-	1.92
Complex I	k	0.0343	0.0691	0.1078	0.0670	0.0178	1.4300	2.4500	-	-	0.0400
	t_0.5_	20.20	10.03	6.43	10.34	38.93	0.48	0.28	-	-	17.33
	t_0.1_	3.07	1.53	0.98	1.57	5.92	0.07	0.04	-	-	2.62
Complex II	k	0.0358	0.0792	0.0581	0.0551	0.0255	0.6294	1.9614	-	-	0.2275
	t_0.5_	19.35	8.75	11.92	12.59	27.23	1.10	0.35	-	-	3.05
	t_0.1_	2.94	1.33	1.81	1.91	4.14	0.17	0.05	-	-	0.46
Tablets	k	0.0339	0.0678	0.0622	0.0752	0.0200	3.2800	3.5300	-	-	0.0250
	t_0.5_	20.44	10.22	11.14	9.22	34.65	0.21	0.20	-	-	27.72
	t_0.1_	3.11	1.55	1.69	1.40	5.27	0.03	0.03	-	-	4.22

**Table 3:** Values of kinetic parameters for irradiated solutions.

Radiation		Parameter	Substance	Complex I	Complex II	Tablets
UV		k	0.5583	0.2583	0.6087	0.3883
		t_0.5_	1.24	2.68	1.14	1.78
		t_0.1_	0.19	0.41	0.17	0.27
Sunlight	Solutions	k	0.0467	0.0450	0.0251	0.0650
		t_0.5_	14.84	15.40	27.61	10.66
		t_0.1_	2.26	2.34	4.20	1.62
	Solid state	k	0.0013	0.0014	0.0074	0.0036
		t_0.5_	533.08	495.00	93.67	192.5
		t_0.1_	81.08	75.29	14.23	29.28
Control						
UV		k	0.0200	0.0183	0.0251	0.0367
		t_0.5_	34.65	37.87	27.61	18.88
		t_0.1_	5.27	5.76	4.20	2.87
Sunlight	Solutions	k	0.0217	0.0200	0.0235	0.0233
		t_0.5_	31.94	34.65	29.54	29.74
		t_0.1_	4.86	5.27	4.49	4.52
	Solid state	k	0.0002	0.0002	0.0001	0.0003
		t_0.5_	3465.00	3465.00	6753.73	2310.00
		t_0.1_	527.00	527.00	1026.22	351.33

**Table 4:** Equations of straight curves, correlation coefficients (r), and activation energy (E_a_) values determined for MTZ solutions.

Solution	Equations	r	E_a_ [J/mol]
Standard substance			
MeOH	logk = 0.9328−0.8352 · 1/T	0.9996	16,183.26
3M HCl	logk = 1.8824−1.0550 · 1/T	0.9480	19,849.41
1M HCl	logk = 0.8641−0.7560 · 1/T	0.9822	15,059.71
0.5M HCl	logk = −0.5991−0.3440 · 1/T	0.9966	6536.26
0.1M HCl	logk = −0.5264−0.3824 · 1/T	0.9996	7371.54
0.1M NaOH	logk = 7.9886−2.9240 · 1/T	0.9956	56,819.34
0.5M NaOH	logk = 7.9068−2.7950 · 1/T	0.9993	54,248.64
H_2_O_2_	logk = 0.9600−0.8033 · 1/T	0.9970	15,435.33
Complex I			
MeOH	logk = 0.5410−0.7374 · 1/T	0.9788	14,227.71
3M HCl	logk = 1.6369−0.9879 · 1/T	0.9057	18,615.45
1M HCl	logk = 1.4218−0.8912 · 1/T	0.9341	17,832.89
0.5M HCl	logk = 2.9912−1.5310 · 1/T	0.9837	29,645.84
0.1M HCl	logk = −0.5641−0.4440 · 1/T	0.9104	8798.15
0.1M NaOH	logk = 9.8273−3.5620 · 1/T	0.9795	69,335.86
0.5M NaOH	logk = 8.8320−3.0810 · 1/T	0.9982	59,659.80
H_2_O_2_	logk = −0.4459−0.3560 · 1/T	0.9265	7171.47
Complex II			
MeOH	logk = 1.3067−1.0011 · 1/T	0.9998	19,425.12
3M HCl	logk = 1.8293−1.0396 · 1/T	0.9311	19,602.68
1M HCl	logk = −0.4581−0.2879 · 1/T	0.9809	5819.35
0.5M HCl	logk = 2.7098−1.4099 · 1/T	0.9350	26,426.23
0.1M HCl	logk = 0.1263−0.6209 · 1/T	0.9966	11,609.09
0.1M NaOH	logk = 7.0361−2.6341 · 1/T	0.9999	50,457.27
0.5M NaOH	logk = 6.6784−2.2956 · 1/T	0.9824	43,777.28
H_2_O_2_	logk = 3.9259−1.6473 · 1/T	0.9922	32,872.42
Tablets			
MeOH	logk = −0.0520−0.5220 · 1/T	0.9818	10,176.46
3M HCl	logk = 0.1622−0.4681 · 1/T	0.9751	8714.21
1M HCl	logk = −0.4612−0.2725 · 1/T	0.9999	5456.29
0.5M HCl	logk = −0.2585−0.3110 · 1/T	0.9933	5835.70
0.1M HCl	logk = 1.1299−1.0030 · 1/T	0.9259	19,153.25
0.1M NaOH	logk = 11.3210−3.9570 · 1/T	0.9930	76,854.51
0.5M NaOH	logk = 9.7085−3.2890 · 1/T	0.9806	62,849.16
H_2_O_2_	logk = −1.3350−0.0934 · 1/T	0.8910	1607.62

## Data Availability

The authors read the data availability statements.

## Supplementary Materials

The following are available online: Table S1. Stability testing of metronidazole, Table S2. Concentration [%)] of MTZ standard substance in solutions of different pH, Table S3. Concentration [%] of MTZ in the form of complex I in solutions of different pH, Table S4. Concentration [%] of MTZ in the form of complex II in solutions of different pH, Table S5. Concentration [%] of MTZ in the solution of tablets at different pH, Table S6. Concentration [%] of MTZ in 3% hydrogen peroxide solutions, Table S7. Concentration [%] of MTZ in UV-treated methanolic solutions (254 nm), Table S8. Concentration [%] of MTZ in methanolic solutions exposed to sunlight irradiation, Table S9. Concentration [%] of MTZ (solid state) exposed to sunlight irradiation, Table S10. Statistical parameters describing the relationship lnc = f(t) for the MTZ solutions, Table S11. Statistical parameters describing the relationship lnc = f(t) for the complex I solutions, Table S12. Statistical parameters describing the relationship lnc = f(t) for the complex II solutions, Table S13. Statistical parameters describing the relationship lnc = f(t) for the tablet solutions, Table S14. Statistical parameters describing the relationship lnc = f(t) for UV irradiated solutions.

## Sample Availability

Samples of the compounds are available from the authors.
