# Supplementary material for: Stability of Metronidazole and Its Complexes with Silver(I) Salts under Various Stress Conditions
Source: Molecules. 2021 Jun 11;26(12):3582. doi: 10.3390/molecules26123582 (PMC8230755; doi:10.3390/molecules26123582)
Supplement: Supplementary file 1 [file molecules-26-03582-s001.zip › molecules-1231815-supplementary.pdf]

molecules

Supplementary Materials

Article:

Stability of Metronidazole and Its Complex Compounds With Silver(I) Salts Under Various Stress Conditions

Małgorzata Starek[1], Monika Dąbrowska[1], Joanna Chebda[1], Dominik Żyro[2] and Justyn Ochocki[2,*]

[1] Department of Inorganic and Analytical Chemistry, Faculty of Pharmacy, Jagiellonian University Medical College, Medyczna 9, 30-688 Kraków, Poland; m.starek@uj.edu.pl (M.S.); monika.1.dabrowska@uj.edu.pl (M.D.); jmalina328@gmail.com (J.C.)
[2] Department of Bioinorganic Chemistry, Chair of Medicinal Chemistry, Faculty of Pharmacy, Medical University of Lodz, Muszyńskiego 1, 90-151 Łódź, Poland; dominik.zyro@umed.lodz.pl (D.Z.); justyn.ochocki@umed.lodz.pl (J.O.)
* Correspondence: justyn.ochocki@umed.lodz.pl; Tel.: +48 42 677 9220

contents:

Table S1. Stability testing of metronidazole;

Table S2. Concentration [%] of MTZ standard substance in solutions of different pH;

Table S3. Concentration [%] of MTZ in the form of complex I in solutions of different pH;

Table S4. Concentration [%] of MTZ in the form of complex II in solutions of different pH;

Table S5. Concentration [%] of MTZ in the solution of tablets at different pH;

Table S6. Concentration [%] of MTZ in 3% hydrogen peroxide solutions;

Table S7. Concentration [%] of MTZ in UV-treated methanolic solutions (254 nm);

Table S8. Concentration [%] of MTZ in methanolic solutions exposed to sunlight irradiation;

Table S9. Concentration [%] of MTZ (solid state) exposed to sunlight irradiation;

Table S10. Statistical parameters describing the relationship lnc=f(t) for the MTZ solutions;

Table S11. Statistical parameters describing the relationship lnc=f(t) for the complex I solutions;

Table S12. Statistical parameters describing the relationship lnc=f(t) for the complex II solutions;

Table S13. Statistical parameters describing the relationship lnc=f(t) for the tablet solutions;

Table S14. Statistical parameters describing the relationship lnc=f(t) for UV irradiated solutions.

Table S2. Stability testing of metronidazole

Analyzed sample	Degradation conditions	Kinetics	Method	Results	Ref.
10 mg of substance	1M HCl in methanol	-	HPTLC	MTZ residue in the range from 87.03 to 100.18%	[4]
	1M NaOH in methanol				
	Dry, warm air; incubation 3h				
	Methanolic solution, incubation under sunlight 8h				
Water-glycol solutions in concentration of 900 mg/100 ml	40°C, humidity 75%, 12 weeks	-	Spectrophotometry λ=277 nm	MTZ is more stable in solutions containing propylene glycol compared to an aqueous solution	[28]
	25°C, humidity 60%, 12 weeks				
1 mg/L metronidazole aqueous solution	UV	Pseudo-first order	HPLC, UV spectrophotometry	MTZ oxidation occurs faster with H_2O_2 compared to photolysis. The degree of drug degradation increases with the increase in Fe^{2+} ion content	[29]
	UV; H_2O_2 (25 and 50 mg)				
	H_2O_2; Fe^{2+}	Second order			
	UV; H_2O_2 (1mg/L); Fe^{2+} 2.94, 5.88 and 11.76 μM; pH=3.5				
Metronidazole monosuccinate aqueous soluton	pH from 1.5 to 10, 60°C	First order	HPLC	The highest stability in the range of pH 3-6	[30]
Metronidazole suspension	30, 37, 45°C	First order	Microbiological method with *B. subtilis*	An increase in temperature leads to increased MTZ degradation	[31]

Tablet (500 mg) and solution for injection (500mg/ 100mL)	Humidity 75% and 4, 20, 40°C, 3 months	Korsmeyera-Peppasa model	Spectop hotomet ry λ=277 nm	The content of MTZ is highest after storage at 20°C for 7 days, while the lowest - at 40°C for 90 days. The tablets stored at 4°C had the highest hardness and the longest degradation time	[32]
	Humidity 75%, 40 and 50°C, 7, 30, 60, 90 days	Pseudo-first order	UV-VIS spectro photom etry	Faster degradation at 50°C. The shelf life was estimated at 25°C using the Q10 value	
Solution for injection (500mg/ 100mL)	37 and 50°C, 4, 50, 90 days	Pseudo-first order	Spectro photom etry, λ=277 nm	The shelf life was estimated (3.8 years) by the factor Q_{10} for 25°C	[33]
Free metronidazole and 3 types of tablet (250 mg)	TG isothermal curves at 413, 423, 433, 443 and 453 K during 240 minutes	First order	TG, DTA, DSC	In 435 K melting piont of MTZ, possible interactions between the drug and excipients in tablets and differences between the tested preparations were demonstrated	[34]
Solutions (4,97·10^{-5} to 14,9·10^{-5}M) in phosphate buffer (pH 6-10) in presence or absence of sodium urate (2,1·10^{-3} to 10,4·10^{-3}M). Citrate and acetate buffer were also tested	Fluorescent light, UV-A, UV-B	Zero order	Spectro photom etry	Degradation depends of pH, type of buffer (the largest in citrate buffer), sodium urate concentration and light source (the largest in fluorescent light)	

Table S2. Concentration [%] of MTZ standard substance in solutions of different pH

t [h]	MeOH	3M HCl	1M HCl	0.5M HCl	0.1M HCl	0.1M NaOH	0.5M NaOH	1M NaOH	3M NaOH
30°C									
0	100.00	100.00	100.00	100.00	100.00	100.00	100.00	100.00	100.00
1	95.58	82.76	84.43	92.17	99.27	95.94	93.48	79.46	71.67
2	89.32	84.58	86.20	89.61	96.54	94.54	91.68	80.53	57.02
3	89.88	77.95	78.93	80.77	93.50	90.44	82.84	59.77	32.09
4	90.02	72.64	77.38	74.61	87.45	88.86	67.20	47.18	29.21
23	70.81	59.46	57.40	65.37	69.41	57.97	31.83	0	0
60°C									
0	100.00	100.00	100.00	100.00	100.00	100.00	100.00	100.00	100.00
1	95.47	93.70	90.92	87.36	99.48	87.61	64.17	15.83	0
2	91.37	90.23	90.92	86.39	94.63	75.76	55.86	0	0
3	90.52	77.31	89.40	85.10	92.76	64.26	47.49	0	0
4	85.83	74.72	71.72	72.64	90.92	50.05	29.22	0	0
23	55.15	21.33	44.70	59.15	61.56	4.38	0	0	0
90°C									
0	100.00	100.00	100.00	100.00	100.00	100.00	100.00	100.00	100.00
1	99.46	80.36	65.86	92.38	92.81	55.69	45.62	0	0
2	93.22	72.62	55.96	79.60	85.01	13.87	3.03	0	0
3	78.48	57.01	49.42	77.07	79.94	0	0	0	0
4	71.43	41.38	43.03	70.02	78.47	0	0	0	0
23	36.60	14.88	22.42	51.94	55.15	0	0	0	0

Table S3. Concentration [%] of MTZ in the form of complex I in solutions of different pH

t [h]	MeOH	3M HCl	1M HCl	0.5M HCl	0.1M HCl	0.1M NaOH	0.5M NaOH	1M NaOH	3M NaOH
30°C									
0	100.00	100.00	100.00	100.00	100.00	100.00	100.00	100.00	100.00
1	96.96	91.60	80.64	98.48	97.94	96.68	72.15	82.34	64.52
2	94.66	88.59	71.58	96.20	95.01	92.55	63.59	74.60	48.81
3	90.51	82.66	63.74	94.02	92.16	85.88	57.82	65.17	34.15
4	85.63	77.02	56.82	90.92	91.37	81.95	49.21	59.54	22.50
23	73.70	62.80	46.53	80.35	79.84	70.81	32.46	0	0
60°C									

0	100.00	100.00	100.00	100.00	100.00	100.00	100.00	100.00	100.00
1	94.28	95.92	74.68	95.58	98.92	92.57	76.33	6.71	0
2	87.36	87.50	64.13	86.49	92.76	79.57	53.49	0	0
3	83.20	80.30	55.68	79.59	90.60	76.02	36.33	0	0
4	81.45	74.44	46.53	73.73	89.30	71.16	26.31	0	0
23	64.72	22.20	36.60	62.80	78.26	15.52	0	0	0
90°C									
0	100.00	100.00	100.00	100.00	100.00	100.00	100.00	100.00	100.00
1	97.35	92.76	78.53	86.30	98.85	24.04	8.68	0	0
2	91.72	58.38	22.89	66.60	98.13	0	0	0	0
3	72.54	39.18	18.71	50.15	92.05	0	0	0	0
4	59.25	27.24	15.41	33.83	86.34	0	0	0	0
23	45.60	20.49	8.41	21.54	66.69	0	0	0	0

Table S4. Concentration [%] of MTZ in the form of complex II in solutions of different pH

t [h]	MeOH	3M HCl	1M HCl	0.5M HCl	0.1M HCl	0.1M NaOH	0.5M NaOH	1M NaOH	3M NaOH
30°C									
0	100.00	100.00	100.00	100.00	100.00	100.00	100.00	100.00	100.00
1	97.75	91.98	80.02	95.71	95.97	95.02	80.87	77.23	60.27
2	93.68	89.73	70.54	88.17	90.04	88.20	71.44	53.18	36.30
3	90.48	80.21	61.08	86.27	86.27	82.34	58.07	38.70	16.01
4	86.90	76.30	54.32	83.74	83.40	80.60	40.53	21.07	0
23	79.62	60.75	40.26	80.21	76.21	59.78	8.26	0	0
60°C									
0	100.00	100.00	100.00	100.00	100.00	100.00	100.00	100.00	100.00
1	90.11	89.23	83.01	88.88	91.19	90.75	52.96	11.11	0
2	82.62	73.09	77.52	78.26	79.54	83.64	23.40	1.40	0
3	78.50	66.82	71.47	72.64	77.54	65.42	7.95	0	0
4	70.32	51.70	48.78	57.41	75.29	45.37	0	0	0
23	62.43	20.11	34.97	35.73	64.44	4.96	0	0	0
90°C									
0	100.00	100.00	100.00	100.00	100.00	100.00	100.00	100.00	100.00
1	75.07	88.13	82.63	78.76	88.42	49.62	6.03	4.07	0
2	66.73	69.05	66.41	66.47	78.11	30.98	1.98	0	0
3	62.46	51.26	50.03	62.33	72.09	19.15	0	0	0
4	58.04	40.09	40.69	37.50	71.86	8.07	0	0	0
23	43.89	16.17	26.27	28.19	55.70	0	0	0	0

Table S5. Concentration [%] of MTZ in the solution of tablets at different pH

t [h]	MeOH	3M HCl	1M HCl	0.5M HCl	0.1M HCl	0.1M NaOH	0.5M NaOH	1M NaOH	3M NaOH
30°C									
0	100.00	100.00	100.00	100.00	100.00	100.00	100.00	100.00	100.00
1	93.42	90.29	75.19	85.27	95.84	95.15	92.33	82.87	62.17
2	89.44	72.90	65.37	66.02	91.37	93.14	87.46	55.70	47.27
3	86.37	65.51	55.54	57.90	90.23	83.73	85.02	34.81	31.30
4	81.45	59.21	52.66	49.90	86.49	80.05	79.27	22.65	21.99
23	67.36	41.68	36.97	30.88	88.33	61.56	26.84	0	0
60°C									
0	100.00	100.00	100.00	100.00	100.00	100.00	100.00	100.00	100.00
1	93.94	95.58	91.37	86.16	93.12	83.48	42.55	7.97	0
2	83.75	71.52	76.62	82.13	88.17	72.64	14.69	0	0
3	80.00	78.26	69.95	76.70	85.59	62.61	3.50	0	0
4	75.19	72.28	62.87	67.87	80.64	45.16	0	0	0
23	59.74	20.49	30.27	44.12	66.69	0	0	0	0
90°C									
0	100.00	100.00	100.00	100.00	100.00	100.00	100.00	100.00	100.00
1	95.58	78.26	47.20	44.63	90.92	3.78	2.93	0	0
2	90.14	54.78	38.47	34.89	83.93	0	0	0	0
3	87.70	42.63	27.79	28.44	79.65	0	0	0	0
4	82.89	34.47	26.58	22.65	78.42	0	0	0	0
23	46.06	21.12	23.96	17.81	63.43	0	0	0	0

Table S6. Concentration [%] of MTZ in 3% hydrogen peroxide solutions

t [h]	Standard substance	Complex I	Complex II	Tablets
30°C				
0	100.00	100.00	100.00	100.00
1	97.90	96.11	96.65	98.98
2	96.66	95.10	95.42	96.71
3	93.73	93.63	93.09	95.58
4	92.75	91.25	90.17	91.61
60°C				
0	100.00	100.00	100.00	100.00
1	99.92	99.63	94.25	99.14
2	97.51	95.67	88.26	98.01
3	93.69	91.84	73.30	95.35

4	86.49	90.02	63.72	90.53
90°C				
0	100.00	100.00	100.00	100.00
1	97.13	94.44	88.97	97.72
2	93.23	90.81	65.41	95.26
3	85.29	89.17	54.07	94.43
4	80.65	85.92	40.26	90.56

Table S7. Concentration [%] of MTZ in UV-treated methanolic solutions (254 nm)

t [h]	Standard substance	Complex I	Complex II	Tablets
0	100.00	100.00	100.00	100.00
1	95.58	94.64	91.34	89.00
2	56.34	80.64	35.35	50.35
3	27.66	56.26	24.49	39.89
4	11.23	47.42	12.04	24.01
5	5.00	32.57	4.77	13.54
6	3.54	21.38	0	9.76
Control				
0	100.00	100.00	100.00	100.00
2	98.00	97.30	97.11	95.18
4	90.72	93.73	88.65	88.77
6	89.40	89.99	86.02	81.00

Table S8. Concentration [%] of MTZ in methanolic solutions exposed to sunlight irradiation

t [h]	Radiation dose [kJ/m^2]	Standard substance	Complex I	Complex II	Tablets
0	0	100.00	100.00	100.00	100.00
1	2324	96.53	91.77	89.44	92.56
2	4746	90.50	90.42	70.12	87.41
3	6677	88.95	88.81	57.36	80.29
4	9030	83.95	86.23	40.07	76.71
5	11259	82.91	80.66	23.50	71.88
6	13828	76.04	76.99	16.51	68.17
Control					
0	0	100.00	100.00	100.00	100.00
2	4746	98.65	98.22	97.68	98.98

4	9030	90.63	92.77	90.03	98.39
6	13828	88.41	89.39	86.87	87.47

Table S9. Concentration [%] of MTZ (solid state) exposed to sunlight irradiation

t [h]	Radiation dose [kJ/m^2]	Standard substance	Complex I	Complex II	Tablets
0	0	100.00	100.00	100.00	100.00
1	2788	100.00	100.00	100.00	100.00
3	7492	99.89	97.85	91.47	98.00
48	120419	98.71	95.42	79.23	87.39
72	182275	96.67	91.03	61.05	78.89
96	242237	89.27	88.07	49.16	70.85
Control					
0	0	100.00	100.00	100.00	100.00
48	120419	100.00	100.00	100.00	100.00
96	242237	98.58	98.62	99.02	97.89

Table S10. Statistical parameters describing the relationship lnc=f(t) for the MTZ solutions

Parameter	r	S_e	a	S_a	b	S_b
30°C						
MeOH	0.9827	0.0103	-0.0300	0.0033	4.6060	0.0080
3M HCl	0.9278	0.0514	-0.0700	0.0162	4.5640	0.0398
1M HCl	0.9091	0.0494	-0.0590	0.0156	4.5640	0.0382
0.5M HCl	0.9850	0.0233	-0.0730	0.0074	4.6120	0.0181
0.1M HCl	0.9548	0.0193	-0.0340	0.0061	4.6260	0.0150
0.1M NaOH	0.9744	0.0126	-0.0300	0.0040	4.6020	0.0098
0.5M NaOH	0.9361	0.0631	-0.0920	0.0200	4.6440	0.0489
1M NaOH	0.9665	0.0878	-0.1810	0.0278	4.6260	0.0680
3M NaOH	0.9832	0.1111	-0.3280	0.0351	4.6080	0.0861
H_2O_2	0.9853	0.0063	-0.0200	0.0020	4.6060	0.0049
60°C						
MeOH	0.9723	0.0162	-0.0370	0.0051	4.6020	0.0126

3M HCl	0.9793	0.0298	-0.0790	0.0094	4.6200	0.0231
1M HCl	0.9120	0.0632	-0.0770	0.0200	4.6460	0.0490
0.5M HCl	0.9316	0.0477	-0.0670	0.0151	4.5880	0.0370
0.1M HCl	0.9794	0.0102	-0.0270	0.0032	4.6140	0.0079
0.1M NaOH	0.9914	0.0413	-0.1710	0.0131	4.6380	0.0320
0.5M NaOH	0.9741	0.1179	-0.2780	0.0373	4.5600	0.0913
1M NaOH	0.9936	0.3715	-2.3050	0.2627	4.7617	0.3391
H_2O_2	0.9333	0.0253	-0.0360	0.0080	4.6300	0.0196
90°C						
MeOH	0.9585	0.0499	-0.0920	0.0158	4.6580	0.0387
3M HCl	0.9848	0.0686	-0.2130	0.0217	4.6360	0.0531
1M HCl	0.9595	0.1066	-0.1990	0.0337	4.4940	0.0826
0.5M HCl	0.9866	0.0275	-0.0910	0.0087	4.6040	0.0213
0.1M HCl	0.9786	0.0250	-0.0650	0.0079	4.5940	0.0193
0.1M NaOH	0.9466	0.8089	-1.5020	0.3617	5.1180	0.6767
0.5M NaOH	0.9819	0.4739	-1.5540	0.2119	4.9660	0.3965
H_2O_2	0.9816	0.0202	-0.0570	0.0064	4.6280	0.0157

r – correlation coefficient; S_e – standard deviation of estimation; a – the slope; S_a – standard deviation of the slope; b – intercept; S_b – standard deviation of the intercept

Table S11. Statistical parameters describing the relationship lnc=f(t) for the complex I solutions

Parameter	r	S_e	a	S_a	b	S_b
30°C						
MeOH	0.9851	0.0121	-0.0380	0.0038	4.6140	0.0094
3M HCl	0.9949	0.0120	-0.0650	0.0038	4.6020	0.0093
1M HCl	0.9885	0.0385	-0.1380	0.0122	4.5680	0.0298
0.5M HCl	0.9944	0.0048	-0.0250	0.0015	4.6140	0.0037
0.1M HCl	0.9884	0.0073	-0.0260	0.0023	4.6060	0.0057
0.1M NaOH	0.9912	0.0126	-0.0520	0.0040	4.6180	0.0098
0.5M NaOH	0.9687	0.0767	-0.1640	0.0242	4.5280	0.0594

1M NaOH	0.9893	0.0342	-0.1270	0.0108	4.5740	0.0265
3M NaOH	0.9978	0.0444	-0.3640	0.0140	4.5900	0.0344
H_2O_2	0.9789	0.0088	-0.0230	0.0028	4.6020	0.0068
60°C						
MeOH	0.9815	0.0196	-0.0550	0.0062	4.6000	0.0152
3M HCl	0.9967	0.0114	-0.0770	0.0036	4.6220	0.0088
1M HCl	0.9880	0.0522	-0.1830	0.0165	4.5540	0.0405
0.5M HCl	0.9963	0.0126	-0.0800	0.0040	4.6220	0.0098
0.1M HCl	0.9774	0.0126	-0.0320	0.0040	4.6100	0.0098
0.1M NaOH	0.9850	0.0288	-0.0900	0.0091	4.6020	0.0223
0.5M NaOH	0.9965	0.0496	-0.3230	0.0157	4.6240	0.0384
H_2O_2	0.9848	0.0097	-0.0300	0.0031	4.6180	0.0075
90°C						
MeOH	0.9776	0.0534	-0.1360	0.0169	4.6660	0.0414
3M HCl	0.9842	0.1144	-0.3480	0.0362	4.7320	0.0886
1M HCl	0.9678	0.2455	-0.5170	0.0776	4.6300	0.1902
0.5M HCl	0.9869	0.0813	-0.2720	0.0257	4.6860	0.0630
0.1M HCl	0.9914	0.0080	-0.0330	0.0025	4.6180	0.0062
H_2O_2	0.9851	0.0121	-0.0380	0.0038	4.5980	0.0094

r – correlation coefficient; S_e – standard deviation of estimation; a – the slope; S_a – standard deviation of the slope; b – intercept; S_b – standard deviation of the intercept

Table S12. Statistical parameters describing the relationship lnc=f(t) for the complex II solutions

Parameter	r	S_e	a	S_a	b	S_b
30°C						
MeOH	0.9264	0.0338	-0.0086	0.0017	4.5671	0.0168
3M HCl	0.9033	0.0851	-0.0185	0.0044	4.5098	0.0423
1M HCl	0.8338	0.1957	-0.0304	0.0101	4.3423	0.0972
0.5M HCl	0.8055	0.0583	-0.0082	0.0030	4.5315	0.0290
0.1M HCl	0.8339	0.0618	-0.0096	0.0032	4.5229	0.0307
0.1M NaOH	0.9517	0.0626	-0.0200	0.0032	4.5316	0.0311

0.5M NaOH	0.9823	0.1907	-0.1029	0.0098	4.4224	0.0947
1M NaOH	0.9878	0.1097	-0.3810	0.0347	4.6900	0.0849
3M NaOH	0.9922	0.1201	-0.6030	0.0537	4.6720	0.1005
H_2O_2	0.9884	0.0073	-0.0260	0.0023	4.6060	0.0057
60°C						
MeOH	0.8082	0.1132	-0.0160	0.0058	4.4647	0.0563
3M HCl	0.9699	0.1582	-0.0649	0.0081	4.4470	0.0786
1M HCl	0.8777	0.2098	-0.0396	0.0108	4.4042	0.1042
0.5M HCl	0.9293	0.1531	-0.0397	0.0079	4.4481	0.0760
0.1M HCl	0.8038	0.1021	-0.0142	0.0053	4.4681	0.0507
0.1M NaOH	0.9939	0.1403	-0.1306	0.0072	4.5752	0.0697
0.5M NaOH	0.9932	0.1561	-0.8440	0.0698	4.7160	0.1306
1M NaOH	0.9998	0.0531	-2.1350	0.0375	4.5883	0.0484
H_2O_2	0.9755	0.0486	-0.1180	0.0154	4.6520	0.0376
90°C						
MeOH	0.8130	0.1799	-0.0259	0.0093	4.3255	0.0894
3M HCl	0.9322	0.2703	-0.0717	0.0139	4.3942	0.1343
1M HCl	0.8449	0.2930	-0.0476	0.0151	4.2786	0.1456
0.5M HCl	0.8229	0.3025	-0.0451	0.0156	4.2930	0.1503
0.1M HCl	0.8586	0.1157	-0.0199	0.0060	4.4464	0.0575
0.1M NaOH	0.9937	0.1232	-0.5990	0.0389	4.5940	0.0954
0.5M NaOH	0.9705	0.6899	-1.9650	0.4879	4.3283	0.6298
H_2O_2	0.9924	0.0525	-0.2320	0.0166	4.6580	0.0407

r – correlation coefficient; S_e – standard deviation of estimation; a – the slope; S_a – standard deviation of the slope; b – intercept; S_b – standard deviation of the intercept

Table S13. Statistical parameters describing the relationship lnc=f(t) for the tablet solutions

Parameter	r	S_e	a	S_a	b	S_b
30°C						
MeOH	0.9921	0.0115	-0.0500	0.0037	4.6000	0.0089

3M HCl	0.9911	0.0339	-0.1380	0.0107	4.6080	0.0262
1M HCl	0.9714	0.0714	-0.1600	0.0226	4.5380	0.0553
0.5M HCl	0.9933	0.0379	-0.1790	0.0120	4.6020	0.0294
0.1M HCl	0.9805	0.0132	-0.0360	0.0042	4.6000	0.0102
0.1M NaOH	0.9850	0.0189	-0.0590	0.0060	4.6200	0.0146
0.5M NaOH	0.9904	0.0145	-0.0570	0.0046	4.5980	0.0112
1M NaOH	0.9907	0.0964	-0.3850	0.0305	4.7140	0.0747
3M NaOH	0.9973	0.0504	-0.3730	0.0159	4.5720	0.0391
H_2O_2	0.9791	0.0080	-0.0210	0.0025	4.6120	0.0062
60°C						
MeOH	0.9913	0.0179	-0.0740	0.0057	4.6040	0.0139
3M HCl	0.9936	0.0179	-0.0860	0.0057	4.6280	0.0139
1M HCl	0.9952	0.0216	-0.1200	0.0068	4.6100	0.0167
0.5M HCl	0.9850	0.0288	-0.0900	0.0091	4.5880	0.0223
0.1M HCl	0.9884	0.0146	-0.0520	0.0046	4.5960	0.0113
0.1M NaOH	0.9831	0.0640	-0.1880	0.0202	4.6300	0.0496
0.5M NaOH	0.9932	0.2070	-1.1140	0.0926	4.7460	0.1732
1M NaOH	0.9957	0.1995	-1.1720	0.0631	4.8040	0.1545
H_2O_2	0.9577	0.0132	-0.0240	0.0042	4.6200	0.0102
90°C						
MeOH	0.9957	0.0080	-0.0470	0.0025	4.6060	0.0062
3M HCl	0.9960	0.0448	-0.2750	0.0142	4.6020	0.0347
1M HCl	0.9346	0.2217	-0.3190	0.0701	4.3800	0.1717
0.5M HCl	0.9446	0.2176	-0.3430	0.0688	4.3720	0.1685
0.1M HCl	0.9689	0.0294	-0.0630	0.0093	4.5840	0.0228
H_2O_2	0.9825	0.0080	-0.0230	0.0025	4.6080	0.0062

r – correlation coefficient; S_e – standard deviation of estimation; a – the slope; S_a – standard deviation of the slope; b – intercept; S_b – standard deviation of the intercept

Table S14. Statistical parameters describing the relationship lnc=f(t) for UV irradiated solutions

Parameter	Substance	Complex I	Complex II	Tablets
r	0.9832	0.9779	0.9834	0.9915
S_e	0.2752	0.1323	0.2392	0.1276
a	-0.6271	-0.2614	-0.6194	-0.4104
S_a	0.0520	0.0250	0.0572	0.0241
b	4.9971	4.7814	4.8719	4.7711
S_b	0.1875	0.0901	0.1731	0.0869
Control				
r	0.9775	0.9978	0.9721	0.9884
S_e	0.0147	0.0039	0.0221	0.0177
a	-0.0215	-0.0185	-0.0290	-0.0365
S_a	0.0033	0.0009	0.0050	0.0040
b	4.6120	4.6130	4.6170	4.6220
S_b	0.0123	0.0032	0.0185	0.0148

r – correlation coefficient; S_e – standard deviation of estimation; a – the slope; S_a – standard deviation of the slope; b – intercept; S_b – standard deviation of the intercept
